# Cytomegalovirus Infection May Trigger Adult-Onset Still's Disease Onset or Relapses

**DOI:** 10.3389/fimmu.2019.00898

**Published:** 2019-04-24

**Authors:** Jinchao Jia, Hui Shi, Mengguo Liu, Tingting Liu, Jieyu Gu, Liyan Wan, Jialin Teng, Honglei Liu, Xiaobing Cheng, Junna Ye, Yutong Su, Yue Sun, Wen Gong, Chengde Yang, Qiongyi Hu

**Affiliations:** ^1^Department of Rheumatology and Immunology, Ruijin Hospital, Shanghai Jiao Tong University School of Medicine, Shanghai, China; ^2^Department of Dermatology, Huashan Hospital, Fudan University, Shanghai, China

**Keywords:** adult-onset Still's disease, virus, CMV, IFI16, AIM2

## Abstract

Previous studies have revealed that several micro-organisms, especially DNA viruses, have been associated with adult-onset Still's disease (AOSD). However, there are no studies on the relationship between the presence of viral infections in AOSD patients with disease occurrence and reactivation. In the present study, we aimed to investigate the presence of antibodies against virus, virus DNA load and nucleic acid sensors in AOSD patients. Anti-viral antibodies were measured by enzyme-linked immunosorbent assay (ELISA) in plasma samples from 100 AOSD patients and 70 healthy controls (HCs). The copy number of cytomegalovirus (CMV) DNA in 100 AOSD patients was detected by PCR. The expression levels of nucleic acid sensors interferon gamma-inducible protein 16 (IFI16) and absent in melanoma 2 (AIM2) in peripheral blood mononuclear cell (PBMC) and skin from AOSD patients and HCs were analyzed by PCR and immunohistochemistry. The levels of antibodies against CMV were significantly higher in AOSD patients compared to HCs. Moreover, the level of anti-CMV IgM antibody was significantly increased in patients with fever, sore throat, arthralgia and rash. CMV DNA was found in plasma of AOSD patients with disease new-onset and relapse. Furthermore, the copy number of CMV DNA significantly increased in patients with fever, sore throat, arthralgia and rash. And the significant associations of the CMV DNA level with the levels of leukocytes, erythrocyte sedimentation rate (ESR), C-reactive protein (CRP) and tumor necrosis factor-α (TNF-α) were observed. Moreover, we found an upregulation of cytoplasmic DNA-sensing receptor IFI16 and AIM2 in PBMC and skin from AOSD patients. In conclusion, our results showed that CMV infection may play a role in the initiation or amplification of inflammatory responses in AOSD.

## Introduction

Adult-onset Still's disease (AOSD) is a rare but clinically well-known systemic inflammatory disease. It is typically characterized by a high spiking fever, evanescent skin rash, arthralgia, sore throat and neutrophilia (1–3). Even though the etiology of AOSD remains unknown, there is evidence that it's triggered by environmental factors with genetic predisposition (4). It has long been suspected that viral infections might contribute to the onset and relapse of AOSD, although definitive evidence has not been presented.

The link between an infectious agent and the development of AOSD has long been discussed (5), and in 1988 research reported that evidence of viral infections was acquired in three of five patients with AOSD, including echovirus 7 and rubella virus (6). Since then, several researchers have reported the occurrence of AOSD after viral infections, including Epstein-Barr virus (EBV), cytomegalovirus (CMV), human herpesvirus 6, influenza virus, parainfluenza viruses, or coxsackie virus (7–9). In addition, AOSD patients often present with high fever, sore throat and rash, just before the initiation of the disease or the relapse. These manifestations were similar to viral infections with infectious danger signal to trigger inflammatory response (10). Studies during the past decade have emphasized the importance of viral infections to AOSD onset, while data available were based on some cases, and a cohort study to determine the existence of viral infections in AOSD is still lacking (11). Therefore, more evidences need to be provided to explore the relationship between viral infections and onset of AOSD with a cohort study.

The cytokine storm of AOSD is critical to the pathogenesis of AOSD, which is activated by innate immune cells. Over-production of pro-inflammatory cytokines is found in AOSD, including TNF-α, interleukin (IL)-1β, IL-18, and IL-6 (2). The starting point of cytokine storm is probably from specific viral danger signals. Host defense against viruses relies partly on receptors that monitor the presence of non-self nucleic acids associated with viral infection (12). Most of the nucleic acid receptors majoring in inducing pro-inflammatory transcription factors have been identified. The RIG-I-like receptor family of RNA sensors include retinoic acid inducible gene I (RIG-I; also known as DExD/H-box helicases 58, DDX58) and melanoma differentiation associated gene 5 (MDA5; also known as IFIH1, interferon induced with helicase C domain 1). The DNA sensors include AIM2 and cyclic GMP-AMP synthetase (cGAS) (13). RIG-I-like receptors detect RNA molecules that are absent from the uninfected host (14). DNA receptors alert the cell to the abnormal presence of non-self nucleic acids in the cytosol (15). The signaling pathways triggered by RNA and DNA receptors can activate NOD-like receptor protein 3 (NLRP3) inflammasome, produce pro-inflammatory cytokines and chemokines (16). To date, the virus signaling pathway in AOSD has not been assessed.

In the present study, we aimed to investigate the association of CMV, herpes simplex virus-1 (HSV-1), herpes simplex virus-2 (HSV-2) and EBV with clinical manifestations by measuring anti-viral IgG and IgM antibodies in plasma from 100 AOSD patients and 70 matched healthy controls. And we determined the relationship between appearance of CMV DNA and disease onset and relapses. Finally, we identified the expression pattern of DNA-sensing receptors in AOSD patients. Our results provided further insight into the role of viral infection in the pathogenesis of AOSD.

## Materials and Methods

### AOSD Patients and Healthy Controls Subjects

A total of 100 AOSD patients (69 active and 31 inactive AOSD patients) and 70 HCs were included in the present study. All patients fulfilled Yamaguchi's criteria after exclusion of those with infectious, neoplastic and autoimmune disorders (17). Patients were considered as having active AOSD if they had fever and/or arthralgia/arthritis and/or any suggestive skin lesions and/or sore throat. New-onset patients were defined as newly diagnosed AOSD patients with the first appearance of the signs or symptoms and no previous treatment of steroid or synthetic disease-modifying antirheumatic drug. All HC subjects were recruited from age- and sex-matched volunteers with no history of autoimmune, rheumatic, or other diseases. Information on demographic and clinical data was entered into a database together with the laboratory test results. The AOSD disease activity of each patient was assessed using a modified Pouchot score (18). The experimental design was approved by the Ethics Committee of Shanghai Jiao Tong University (identifier 2016-62), and all the participants provided informed consent. All plasma and serum samples were stored at −80°C immediately after collection.

### Detection of Anti-viral IgG and IgM Antibodies in Plasma by ELISA

Anti-viral (CMV, HSV1, HSV2, EBV) antibodies in plasma were analyzed by ELISA kits (Trinity Biotech, New York, USA) according to the manufacturers' instructions. The 96-well plates were incubated with human plasma (1:81) at 37°C for 2 h and washed five times with PBS plus 0.05% Tween-20. The negative control, positive control, standards were added to confirm the validity of the experiment. Secondary horseradish peroxidase (HRP)-conjugated goat anti-human IgM or IgG monoclonal antibodies were added to each well. After five washes with PBS plus 0.05% Tween 20, 100 μL of tetramethybenzidine substrate solution (TMB) was added, and the samples were incubated at room temperature. The reaction was terminated by the addition of 100 μL of 2 N H_2_SO_4_/well, and optical density (OD) was measured at 450 nm. The cutoff values were equal to the average OD value of standards multiplied by the cutoff factor.

### Real-Time PCR for CMV Viral Load Measurements

DNA from 50 μL plasma was amplified using the Real-time PCR system instrument with Human Cytomegalovirus Nucleic Acid Quantitative Detection Kit (DAAN Gene Co., Ltd., China) according to the manufacturers' instructions. Fifty microliter plasma samples were mixed with nucleic acid extraction solution to purify CMV DNA. Two microliter CMV DNA standard or DNA sample as well as 3 μL Taq DNA polymerase were mixed with 40 μL HCMV-PCR reaction solution for viral DNA amplification and quantitation. CMV-positive and negative controls were run in each PCR assay. The enzyme was activated at 93°C for 2 min, followed by 40 cycles at 93°C for 5 s and 57°C for 45 s. The viral load of CMV was expressed with copies/mL.

### Determination of DNA-Sensors

#### Detection of DNA-Sensors Expression Using Quantitative PCR (qPCR)

Venous blood samples were obtained from untreated, active and hospitalized AOSD patients who had available samples at the time of inclusion (*N* = 20) and healthy controls (*N* = 21). Briefly, PBMC was isolated by density gradient centrifugation on Lymphoprep (Axis-Shield, Dundee, UK) according to the manufacturer's instructions (19). The collected cells were used for RNA extraction using RNAeasy columns (Qiagen, Hilden, Germany), and cDNA was prepared by PrimeScript RT Master Mix transcription kit (TaKaRa, Tokyo, Japan). The mRNA expression levels of DNA-sensors expression were determined by SYBR green master mix (Takara). To standardize mRNA expression levels of DNA-sensors, the mRNA levels of the housekeeping gene glyceraldehyde 3-phosphate dehydrogenase (GAPDH) were also determined in parallel for each sample. Relative mRNA expression was calculated as described previously.

#### Detection of IFI16 and AIM2 Expression by Immunohistochemical Analyses

Skin biopsies from AOSD patients with typical skin rash during fever spikes (*N* = 4) and healthy controls (*N* = 4) were analyzed for protein expression (see Supplemental Files for detailed protocol) by immunohistochemistry using the following antibodies: rabbit anti-IFI16 (CST, USA) and rabbit anti-AIM2 (CST, USA). The reaction was then visualized under light microscopy (BX51 Olympus, Japan).

### MSD for Detecting TNF-α, IL-1β, IL-6, and IL-18

Serum TNF-α, IL-1β, IL-6, and IL-18 were measured by the Meso Scale Discovery electrochemiluminescence assay (MSD, Rockville, MD, USA) according to manufacturer's instructions.

### Statistical Analysis

All data were statistically analyzed using the SPSS version 20.0 software (SPSS Inc., Chicago, IL, USA). Quantitative data are expressed as the means ± SD. Data between two groups with a Gaussian distribution were analyzed using an unpaired *t*-test, while non-parametric data were assessed using the Mann-Whitney *U* test. Data among three groups or more were analyzed using one-way analysis of variance (ANOVA) or Wilcoxon rank-sum test. Spearman correlation analysis was performed to test whether anti-CMV antibody levels and the copy number of CMV DNA are correlated with clinical variables. *P*-values <0.05 were considered statistically significant.

## Results

### Higher Antibody Levels Against Cytomegalovirus (CMV) in AOSD

In order to determine whether AOSD patients have an immune response to DNA virus, including CMV, HSV-1, HSV-2, and EBV, which were the relatively common DNA viruses in clinical practice, we next employed ELISA to test. Plasma from 100 AOSD patients (69 active and 31 inactive patients) and 70 healthy controls (HCs) were collected. The clinical characteristics of these subjects in each group are detailed in Table 1.

**Table 1:** Demographic and clinical characteristics of individuals with AOSD.

**Characteristics**	**Active AOSD (*N* = 69)**	**Inactive AOSD (*N* = 31)**	**HCs (*N* = 70)**
Age (years)	38.7 ± 14.9	34.9 ± 11.5	37.3 ± 13.8
Gender (F/M)	48/21	25/6	49/21
Fever	62(89.9)	0	
Evanescent rash	60(86.7)	3(9.7)	
Sore throat	46(66.7)	3(9.7)	
Arthralgia	55(79.7)	2(7.7)	
Pneumonia	21(30.4)	0	
Pleuritis	14(20.3)	0	
Pericarditis	8(11.6)	0	
Hepatomegaly	10(14.5)	0	
Splenomegaly	26(37.7)	0	
Lymphadenopathy	40(58)	0	
Myalgia	26(37.7)	0	
Hemoglobin, g/L	113 ± 20.7	118 ± 22.6	
Leukocytes, 10^9^/L	16.4 ± 6.7	8.8 ± 2.3	
Platelets, 10^9^/L	290.0 ± 105.6	254.5 ± 110.1	
ALT (U/L)	107.4 ± 1,245.2	23.6 ± 15.2	
AST (U/L)	76.5 ± 129.9	20.9 ± 10.2	
Ferritin, ng/mL	>2,000	342.4 ± 458.0	
ESR, mm/h	70.8 ± 29.2	15.4 ± 11.7	
CRP, mg/L	59.7 ± 53.7	6.3 ± 8.3	
Systemic score	7.3 ± 2.6	0.5 ± 0.8	

*All values are presented as numbers (with percentage) or mean ± SD (standard deviation)*.

Among the 69 active AOSD patients, common manifestations included spiking fever (62, 89.9%), evanescent rash (60, 86.7%), sore throat (46, 66.7%), and arthralgia (55, 79.7%). Hepatomegaly, splenomegaly and lymphadenopathy were noted in 10 (14.5%), 26 (37.7%), and 26 (37.7%) patients, individually. There were no significant differences in terms of mean age or sex distribution between the AOSD patients and HCs (*P* = 0.9338 and *P* = 0.6690).

**Figure 1:**
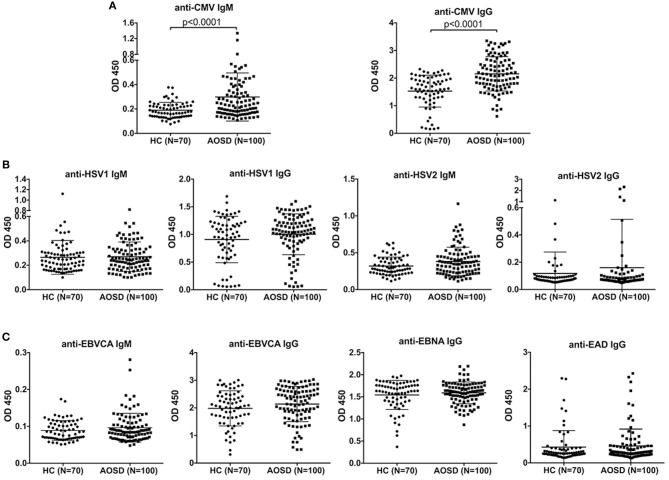
Anti-virus antibody levels in AOSD patients (*N* = 100) and HCs (*N* = 70). **(A)** Comparison of antibody levels of anti-CMV IgM and IgG in AOSD patients and HCs. **(B)** Comparison of IgM and IgG antibody levels against HSV1 and HSV2 in AOSD patients and HCs. **(C)** Comparison of anti-EBVCA IgM, anti-EBVCA IgG, anti-EBNA IgG, and anti-EAD IgG levels in AOSD patients and HC. Each symbol represents an individual patient with AOSD and a HC. The data represent the mean ± SD by Student's *t*-test. CMV, Cytomegalovirus; HSV, Herpes simplex virus; EBVCA, Epstein-Barr virus capsid antigen; EBNA, Epstein-Barr virus nuclear antigen; EAD, Epstein-Barr virus early antigen diffuse.

As shown in Figure 1A, the antibody levels of anti-CMV IgM and IgG were significantly higher in AOSD patients than in HCs (both *P* < 0.0001). The positive rate of anti-CMV IgM antibodies was 5% in AOSD patients, 0 in healthy controls. And the positive rate of anti-CMV IgG antibodies was 93% and 90% in AOSD patients and HCs. Moreover, the levels of anti-virus antibody against HSV1, HSV2, and EBV were up-regulated in AOSD patients compared to HCs, though there was no significant difference (Figures 1B,C). These results suggest that AOSD patients have more enhanced immune response to CMV with higher levels of anti-CMV IgG and IgM antibodies.

### Higher Levels of Anti-CMV Antibodies in Active AOSD

**Figure 2:**
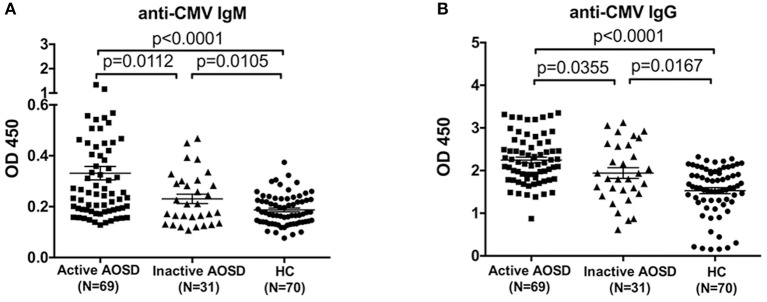
Anti-CMV antibody levels in AOSD patients with diverse disease activity and HCs. **(A)** Plasma levels of anti-CMV IgM antibody in active AOSD (*N* = 69), inactive AOSD (*N* = 31) and HCs (*N* = 70). **(B)** Plasma levels of anti-CMV IgG antibody in active AOSD, inactive AOSD and HCs. The data represent the mean ± SD by Student's *t*-test.

Furthermore, we assessed the levels of anti-CMV antibody in AOSD patients with diverse disease activity. Remarkably, the plasma levels of anti-CMV IgM and IgG antibodies were significantly up-regulated in AOSD patients with active disease (*N* = 69) compared to those with inactive disease (*N* = 31) (Figure 2, *P* = 0.0112 and *P* = 0.0355). Moreover, anti-CMV IgM and IgG levels differed significantly, with higher antibody levels detected in patients with active AOSD compared to HCs (Figure 2, both *P* < 0.0001). And the levels of anti-CMV antibody were also significantly higher in inactive AOSD compared to HCs (Figure 2, *P* = 0.0105 and *P* = 0.0167). Taken together, our data indicate that both IgM and IgG antibodies against CMV are associated with the disease activity of AOSD.

### Anti-CMV Antibodies in AOSD Were Associated With Clinical Manifestations

**Figure 3:**
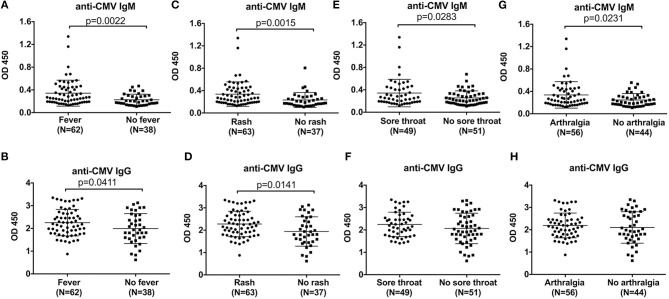
Comparison of the anti-CMV antibody levels in AOSD patients with different clinical manifestations. The levels of anti-CMV IgM and IgG antibody in AOSD patients with or without fever **(A,B)**, rash **(C,D)**, sore throat **(E,F)**, and arthralgia **(G,H)**. The data represent the mean ± SD by Student's *t*-test.

We next assessed the correlations between the levels of anti-CMV antibody and clinical manifestations. Compared with AOSD patients without high spiking fever, the levels of anti-CMV IgM and IgG antibody were significantly higher in AOSD patients with fever (Figures 3A,B, *P* = 0.0022 and *P* = 0.0411). In addition, our results showed that patients with sore throat had higher level of anti-CMV IgM and IgG antibody (Figures 3C,D, *P* = 0.0015 and *P* = 0.0141). And in the presence of sore throat, the level of anti-CMV IgM antibody was significantly increased in AOSD patients (Figures 3E, *P* = 0.0283). The level of anti-CMV IgG tended to be higher in AOSD patients with sore throat (Figure 3F). Furthermore, the level of anti-CMV IgM antibody was significantly higher in patients with arthralgia than in patients without arthralgia (Figure 3G, *P* = 0.0231). In AOSD patients with arthralgia, the level of anti-CMV IgG antibody didn't show significant difference compared with those without arthralgia (Figure 3H). Moreover, the difference of anti-CMV antibody in patients with other clinical manifestations, including pneumonia, pleuritis, pericarditis, hepatomegaly, splenomegaly, lymphadenopathy and myalgia didn't reach statistic difference (Supplementary Figure 1). Taken together, AOSD patients have an immune response to CMV, leading to the onset and relapses, as Yamaguchi's criteria includes the clinical manifestations of high spiking fever, skin rash, arthralgia and sore throat.

### CMV Viral Load Was Higher in Reactive AOSD

**Figure 4:**
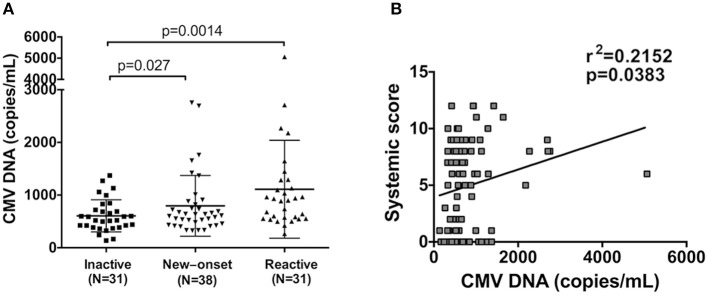
Detection of CMV DNA copy number in AOSD patients. **(A)** Comparison of CMV DNA copies in patients with inactive (*N* = 31), new onset (*N* = 38) and active AOSD (*N* = 31). **(B)** Correlation between CMV DNA copy number and AOSD disease systemic score. The data represent the mean ± SD by Student's *t*-test in **(A)**. The correlations were evaluated with Spearman's non-parametric test in **(B)**. *p* < 0.05 represents a significant difference.

Due to the high level of anti-CMV antibodies in AOSD patients, we further aimed to detect the copy number of CMV DNA in 100 AOSD patients. To exclude the influence of treatment on CMV viral load, 69 active AOSD patients were stratified into 38 new-onset patients and 31 reactive patients. Compared with inactive AOSD patients (*N* = 31), significantly higher copy number of CMV DNA in plasma was observed in new-onset patients (*N* = 38) (Figure 4A, *P* = 0.027). Further comparison was performed between AOSD patients with inactive and reactive disease. Remarkably, compared with AOSD patients with inactive disease, there was significantly more copy number of CMV DNA in plasma in reactive disease (*N* = 31) (Figure 4A, *P* = 0.0014), indicating the presence of CMV infection in AOSD patients with higher disease activity. And then we investigated whether high copy number of CMV DNA was associated with increased systemic score. It revealed that the copy number of CMV DNA was positively associated with systemic score, with a low correlation coefficient (Figure 4B, r^2^ = 0.2152, *P* = 0.0383). In conclusion, CMV infection was more frequent in AOSD patients with disease onset and exacerbation.

### CMV Viral Load Was Associated With Clinical Manifestation

Due to high level of anti-CMV IgM antibody in AOSD patients with fever, rash, sore throat and arthralgia, we further analyzed the difference of CMV DNA copy number in patients with and without these clinical manifestations. As shown in Table 2, higher copy number of CMV DNA was observed in patients with fever (*P* = 0.0039), rash (*P* = 0.0462), sore throat (*P* = 0.0006) and arthralgia (*P* = 0.0156). And the difference of CMV viral load in patients with other clinical manifestations, including pneumonia, pleuritis, pericarditis, hepatomegaly, splenomegaly, lymphadenopathy and myalgia didn't reach statistical difference. Thus, CMV infections would be related with AOSD as Yamaguchi's criteria includes the clinical manifestations of high spiking fever, arthralgia, sore throat and arthralgia.

**Table 2:** Comparison of CMV DNA copy number according to disease manifestations in 100 patients with adult-onset Still's disease.

**Manifestations**	**CMV DNA copy number (copies/mL)**		***P*-value**
Fever	(+), *N* = 62	968.9 ± 100.9	0.0039
	(−), *N* = 38	614.7 ± 50.68	
Skin rash	(+), *N* = 63	944.1 ± 100.1	0.0462
	(−), *N* = 37	647.4 ± 53.89	
Sore throat	(+), *N* = 49	1024 ± 118.1	0.0006
	(−), *N* = 51	652.5 ± 58.64	
Arthralgia	(+), *N* = 57	972.7 ± 108.1	0.0156
	(−), *N* = 43	650.9 ± 53.68	
Hepatomegaly	(+), *N* = 10	964.3 ± 136.5	0.053
	(−), *N* = 90	819.9 ± 73.41	
Splenomegaly	(+), *N* = 26	853.5 ± 101.7	0.3428
	(−), *N* = 74	827.6 ± 84.2	
Lymphadenopathy	(+), *N* = 40	819.7 ± 86.51	0.6451
	(−), *N* = 60	844.1 ± 97.05	
Pneumonia	(+), *N* = 21	975.0 ± 162.6	0.4734
	(−), *N* = 79	796.9 ± 73.63	
Pleuritis	(+), *N* = 14	918.1 ± 141.8	0.1749
	(−), *N* = 86	820.7 ± 75.13	
Pericarditis	(+), *N* = 8	879.4 ± 159.5	0.4401
	(−), *N* = 92	830.4 ± 72.16	
Myalgia	(+), *N* = 26	916.1 ± 123.6	0.1541
	(−), *N* = 74	805.6 ± 80.34	

*CMV DNA copy number are shown as mean ± SD, and differences between two groups were analyzed using the Mann-Whitney U test for nonparametric data*.

**Figure 5:**
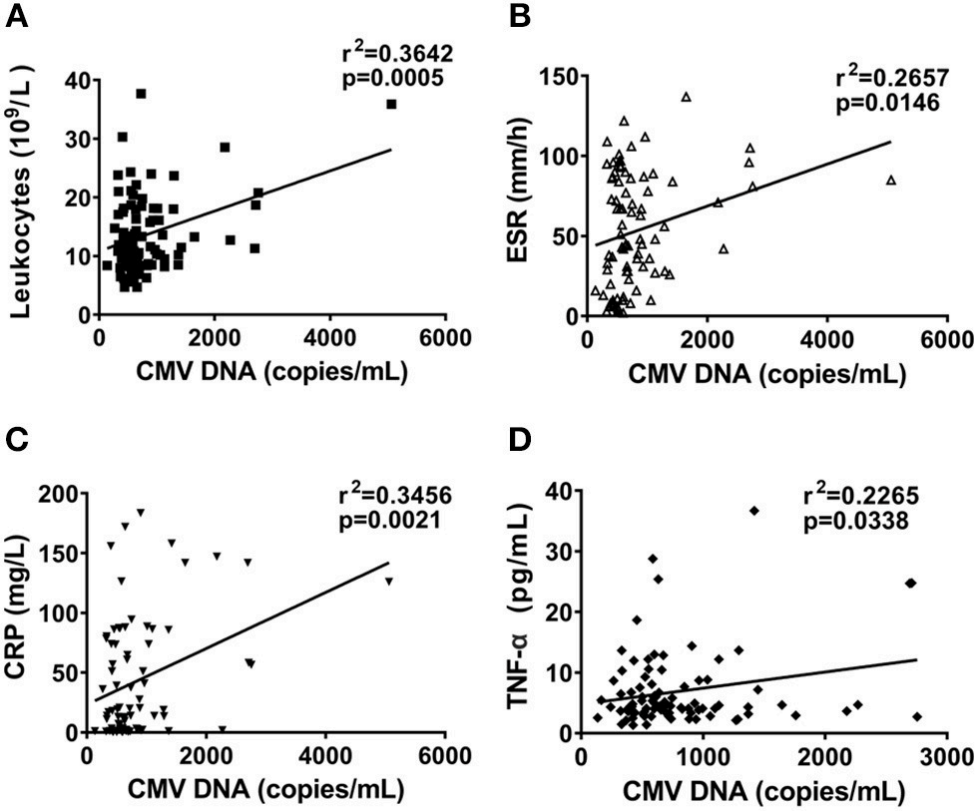
Correlation between CMV DNA copy number and inflammatory index in AOSD patients. Correlation between CMV DNA copy number and leukocytes **(A)**, ESR **(B)**, and CRP **(C)**, and TNF-α levels **(D)**. The correlations were evaluated with Spearman's non-parametric test. *p* < 0.05 represents a significant difference.

We next measured the correlation coefficient of CMV DNA levels with inflammatory index, including leukocytes, ESR, and CRP. The copy number of CMV DNA were positively associated with leukocytes (Figure 5A, r^2^ = 0.3642, *P* = 0.0005) and ESR (Figure 5B, r^2^ = 0.2657, *P* = 0.0146), as well as CRP (Figure 5C, r^2^ = 0.3456, *P* = 0.0021). Furthermore, the significant association was found between the CMV DNA copy number and TNF-α levels (Figure 5D, r^2^ = 0.2265, *P* = 0.0338). We found that CMV DNA copy number showed no significant correlation with IL-1β, IL-6, and IL-18 in AOSD patients (data was not shown). Taken together, the results indicate that CMV DNA may play a role in the inflammatory response in AOSD.

### DNA-Sensing PRRs Were Upregulated in AOSD

**Figure 6:**
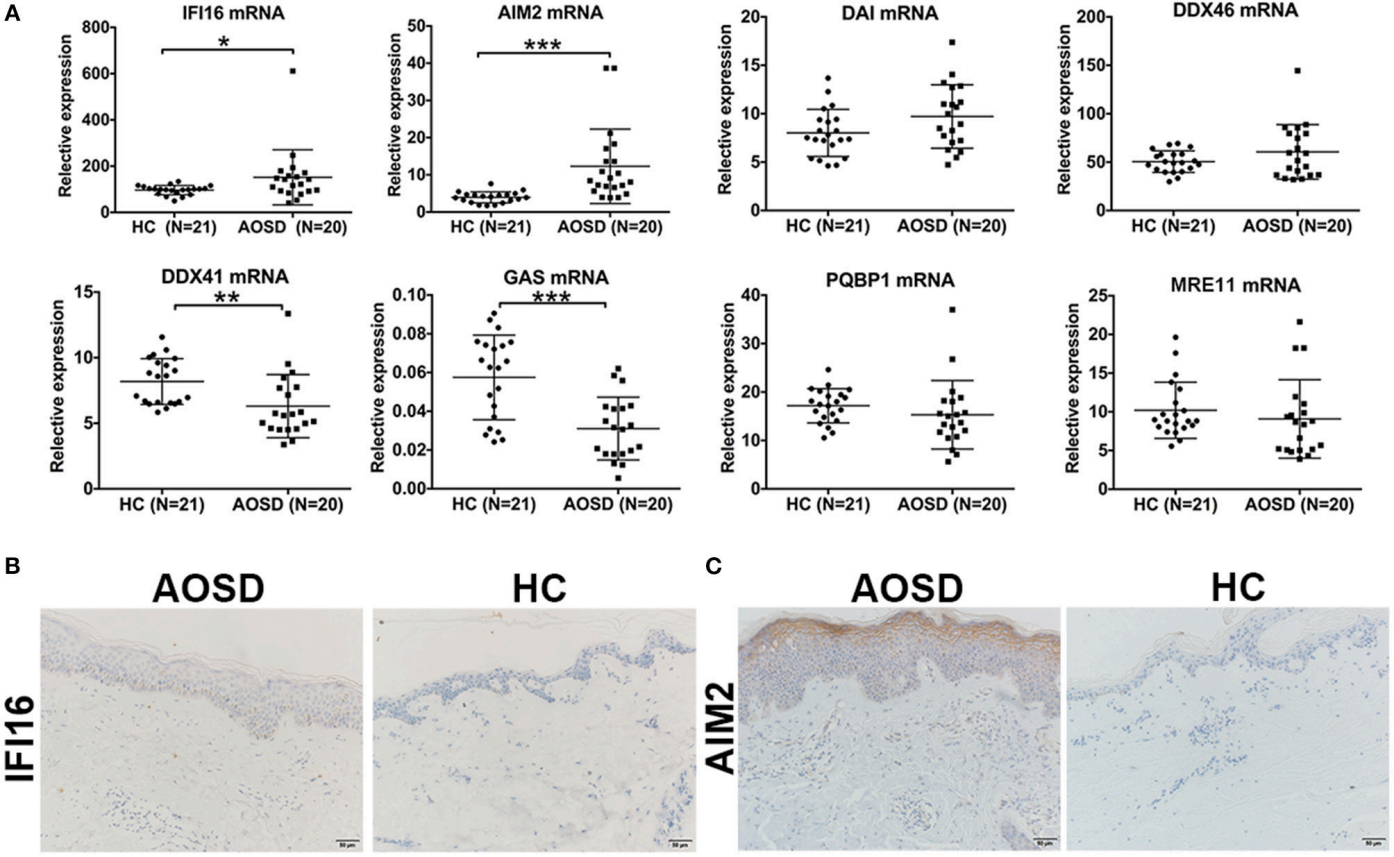
Detection of DNA sensors in AOSD patients. **(A)** Comparison of diverse DNA-sensing receptors in AOSD patients and HCs. **(B,C)** Skin lesion tissue from an AOSD patient and a HC were stained with antibodies specific for IFI16 **(B)** and AIM2 **(C)**. Representative examples of immunohistochemical data (AOSD, *N* = 4; HCs, *N* = 4) are shown. Scar bar: 50 μm. Original magnification: 200×.

To demonstrate the pathogenetic role of viral infections in AOSD, we first detected the nucleic acid receptors that initiate virus sensing pathway. We found a significant upregulation of IFI16 (Figure 6A, *P* < 0.05) in PBMC of AOSD compared with HC PBMC. The level of AIM2 expression had an increasing tendency in AOSD patients with statistical significance (Figure 6A, *P* < 0.001). The level of DNA-dependent activator of IFN-regulatory factors (DAI) and DDX46 has an increasing tendency without any significant difference (Figure 6A). In contrast, other DNA-sensing receptors, DDX41 and GAS were significantly downregulated in AOSD patients compared with HCs (Figure 6A, *P* < 0.01 and *P* < 0.001, receptively). Additionally, polyglutamine binding protein 1 (PQBP1) and meiotic recombination 11 (MRE11) tended to be lower in AOSD patients compared with HCs (Figure 6A). In conclusion, our data suggest that DNA-sensing receptors IFI16 and AIM2 were higher in AOSD patients.

To further assess protein expression of IFI16 and AIM2 in AOSD patients, we assessed the expression of IFI16 and AIM2 in AOSD skin biopsies (*N* = 4) and healthy controls (*N* = 4). Figures 6B,C showed a representative staining for AOSD skin. The staining pattern for IFI16 and AIM2 was most prominent in patients with AOSD. Based on these results, we hypothesize that DNA viral infection may play a role in AOSD patients.

## Discussion

A number of studies have addressed the possible role of viral infections in the pathogenesis of AOSD, while to date, the evidence and the pathogenic role of viral infections in AOSD is still to be determined. A potential role for viral infection in the pathogenesis of autoimmune disease has long been an attractive hypothesis, in part based on clinical manifestations that mimic acute or chronic viral infection (20). The concept that environmental factors including infections trigger disease in individuals with certain genetic backgrounds is broadly recognized (21). The presence of elevated plasma anti-viral antibody and virus DNA in blood and tissue of patients with lupus and other systemic autoimmune diseases, including primary Sjogren's syndrome (SS) (22), rheumatoid arthritis (RA) (23), and dermatomyositis (24) are consistent with a viral trigger, indicating that autoimmune disease can be influenced by viral infections. We hypothesized that viruses, which represent an endogenous source of ligands for nucleic acid sensors, promote a host microenvironment supportive of immune dysfunction, autoimmunity, and inflammation, similar to the immunopathological characteristic of some chronic viral infections (25, 26).

To pursue this possibility, we studied the expression pattern of nucleic acid sensors in AOSD patients. We found that the expression of DNA-sensing receptors significantly increased in PBMC and skin biopsies from AOSD patients, indicating that non-self nucleic acids may present in AOSD. We then used ELISA analysis to detect anti-viral antibodies in plasma samples from 100 AOSD patients and 70 healthy controls. We demonstrated that anti-CMV IgM and IgG antibodies were significantly higher in AOSD patients compared to HCs. Moreover, elevated levels of anti-CMV IgM antibodies were found in patients with fever, sore throat, arthralgia and rash, which are typical clinical manifestations of AOSD. After identifying CMV DNA in plasma of AOSD patients with disease new-onset and relapse, we further demonstrated that CMV infections play a role in the initiation or amplification of the inflammatory responses in AOSD.

Much attention has been drawn potential pathogenetic role of diverse bacterial and viral pathogens in AOSD, particularly virus, including CMV, EBV, rubella virus and human herpesvirus 6 (4). The results of these studies, taken in aggregate, are suggestive of a viral contribution to AOSD, though findings offer little insight into potential mechanisms, and a consistent association with a specific exogenous virus as an etiologic agent has not emerged in AOSD. Human CMV, a ubiquitous beta-herpes virus, has been reported to be associated with several autoimmune diseases (27–29). While, there are insufficient data to directly implicate CMV infection in the onset and relapse of AOSD. Several characteristics of CMV raised our interest in investigating this DNA virus as a candidate trigger of an innate immune response and autoimmunity. First CMV DNA was positively correlated with the count of leukocytes in AOSD patients. Neutrophils, the first line of innate immune defense against infection, are the primary effector cells in the pathogenesis of AOSD (30). Recent data demonstrate a profound survival response and delayed apoptosis in neutrophils exposed to CMV by secreting pro-survival secretome (31), further supporting the hypothesis that CMV infection may trigger inflammatory response of neutrophils. Second CMV-exposed neutrophils release factors that enhance monocyte recruitment, facilitating the inflammatory response (31). The correlation between CMV DNA and acute phase protein (ESR and CRP) in our study suggests CMV infections as underlying contributors to the cytokine storm in AOSD. Third it has been established that inflammatory monocyte/macrophages egress from the bone marrow and into the peripheral blood during CMV infection in a C-C motif chemokine receptor (CCR)2-depedent manner (32), further supporting investigation of the mechanism for CMV in the immunopathology of AOSD by activating neutrophils and macrophages. However, a clear association between CMV seroprevalence and disease has thus far been difficult to establish, because CMV is widespread, and then there is indeed a need for further follow-up study of AOSD patients enrolled in our study. Besides, the relatively low correlation coefficients between CMV DNA load and inflammatory index indicated that other endogenous or exogenous trigger factors may be involved in the pathogenesis of AOSD, which should be explored in a large cohort of AOSD patients in the future. Moreover, the direct relationship of CMV in inducing AOSD needs further investigation *in vivo*.

Since CMV belongs to DNA virus, we hypothesized that it might be recognized by cytoplasmic DNA sensors and potentiate an innate immune response. Elevated expressions of IFI16 and AIM2 in PBMC and skin biopsy from AOSD patients lead to a hypothesis that DNA virus might represent an endogenous source of ligands. Studies labeled IFI16 and AIM2 as a new family of innate DNA sensors “AIM2-like receptors (ALRs)” (33). The ability of IFI16 and AIM2 to induce the inflammasome (34, 35) also draws attention to a potential role of IFI16 and AIM2 in AOSD. Infected with herpesvirus, IFI16 interacts with the adaptor molecule ASC and procaspase-1 to assemble a functional inflammasome (36). And AIM2 has been demonstrated to trigger caspase-1 dependent signaling and innate immunity to DNA viruses, particularly mouse CMV (37). Thus, our findings suggest a potential role of CMV infections in AOSD by activating two DNA sensors, IFI16, and AIM2.

In conclusion, levels of anti-CMV IgM and IgG antibodies were higher in AOSD patients, and increased copy number of CMV DNA was found in patients with new-onset and reactive AOSD. Moreover, we found an upregulation of cytoplasmic DNA-sensing receptor IFI16 and AIM2 in PBMC and skin from AOSD patients, further supporting the hypothesis that CMV infections exist in AOSD. In addition, anti-CMV IgM antibody level and CMV DNA copy number were significantly increased in patients with fever, sore throat, arthralgia and rash. And the significant association of the CMV DNA level with the levels of leukocytes, ESR, CRP and TNF-α was observed, suggesting that CMV infections may play a role in the inflammatory response in AOSD. Taken together, these data provide some evidence that CMV infections might contribute to the onset and exacerbations of AOSD.

## Ethics Statement

The study was performed in accordance with the Declaration of Helsinki and the principles of Good Clinical Practice. Biological samples were obtained under a protocol approved by the Institutional Research Ethics Committee of Ruijin Hospital (ID: 2016-62), Shanghai, China. All subjects signed written informed consent.

## Author Contributions

JJ and HS participated in RNA extraction, qRT-PCR, and wrote the manuscript. ML analyzed the results of skin biopsy. TL helped to revise the manuscript. JG and LW collected the clinical samples and data. JT and HL prepared the figures. XC and JY prepared the tales. YutS and YueS performed statistical analysis. WG prepared the supplementary figures. CY designed the experiments and revised the manuscript. QH designed experiments, performed statistical analysis, and revised the manuscript. All authors read and approved the final manuscript.

### Conflict of Interest Statement

The authors declare that the research was conducted in the absence of any commercial or financial relationships that could be construed as a potential conflict of interest.

## Supplementary Material

Supplementary Figure 1 Comparison of the anti-CMV IgM antibody levels in AOSD patients with different clinical manifestations. The levels of anti-CMV IgM antibody in AOSD patients with or without pneumonia **(A)**, pleuritis **(B)**, pericarditis **(C)**, hepatomegaly **(D)**, splenomegaly **(E)**, lymphadenopathy **(F)**, and myalgia **(G)**. The data represent the mean ± SD by Student's *t*-test.

Supplementary Figure 2 Comparison of the anti-CMV IgG antibody levels in AOSD patients with different clinical manifestations. The levels of anti-CMV IgG antibody in AOSD patients with or without pneumonia **(A)**, pleuritis **(B)**, pericarditis **(C)**, hepatomegaly **(D)**, splenomegaly **(E)**, lymphadenopathy **(F)**, and myalgia **(G)**. The data represent the mean ± SD by Student's *t*-test.

## Abbreviations

AOSD: Adult-onset Still's disease
HC: Healthy control
SS: Sjogren's syndrome
RA: Rheumatoid arthritis
CMV: Cytomegalovirus
HSV: Herpes simplex virus
EBV: Epstein-Barr virus
IFI16: Interferon gamma-inducible protein 16
AIM2: absent in melanoma 2
ELISA: Enzyme-linked immunosorbent assay
qPCR: Quantitative polymerase chain reaction
PBMC: peripheral blood mononuclear cell
GAPDH: Glyceraldehyde 3-phosphate dehydrogenase
ESR: Erythrocyte sedimentation rate
CRP: C-reactive protein
TNF-α: Tumor necrosis factor-α
IL: Interleukin
RIG- I: Retinoic acid inducible gene I
IFIH1: Interferon induced with helicase C domain 1
DDX: DExD/H-box helicases
cGAS: cyclic GMP-AMP synthetase
NLRP3: NOD-like receptor protein 3
DAI: DNA-dependent activator of IFN-regulatory factors
HRP: Horseradish peroxidase
TMB: Tetramethybenzidine substrate solution
OD: Optical density
CCR: C-C motif chemokine receptor
ALR: AIM2-like receptors
PQBP1: Polyglutamine binding protein 1
MRE11: meiotic recombination 11.
