# Supplementary figures and images for: Cytomegalovirus Infection May Trigger Adult-Onset Still's Disease Onset or Relapses
Source: Front Immunol. 2019 Apr 24;10:898. doi: 10.3389/fimmu.2019.00898 (PMC6491741; doi:10.3389/fimmu.2019.00898)

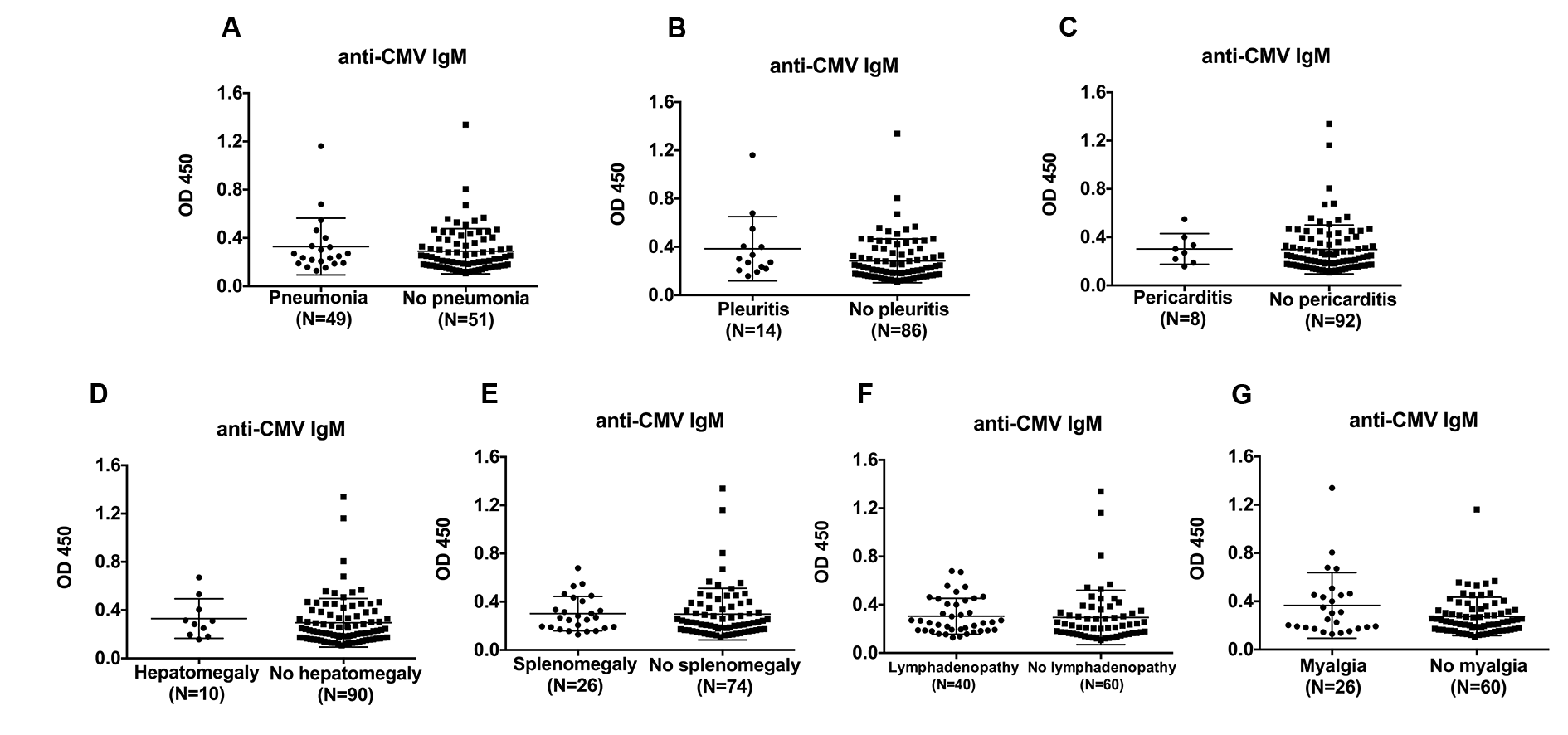

Supplement: Supplementary Figure 1 — Comparison of the anti-CMV IgM antibody levels in AOSD patients with different clinical manifestations. The levels of anti-CMV IgM antibody in AOSD patients with or without pneumonia (A), pleuritis (B), pericarditis (C), hepatomegaly (D), splenomegaly (E), lymphadenopathy (F), and myalgia (G). The data represent the mean ± SD by Student's t-test. [file Image_1.TIF]

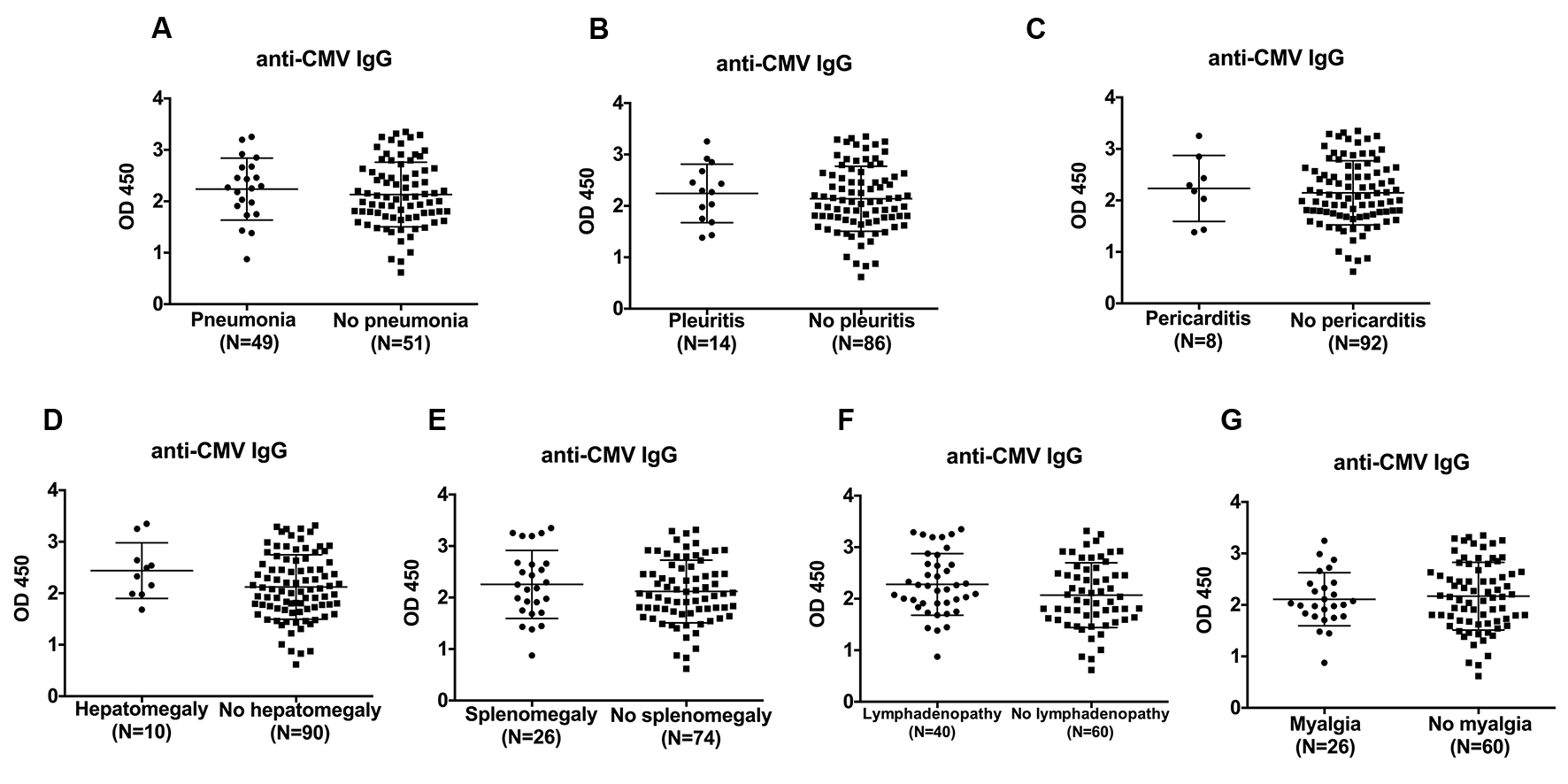

Supplement: Supplementary Figure 2 — Comparison of the anti-CMV IgG antibody levels in AOSD patients with different clinical manifestations. The levels of anti-CMV IgG antibody in AOSD patients with or without pneumonia (A), pleuritis (B), pericarditis (C), hepatomegaly (D), splenomegaly (E), lymphadenopathy (F), and myalgia (G). The data represent the mean ± SD by Student's t-test. [file Image_2.TIF]
